# Hybrid deep learning models with multi-classification investor sentiment to forecast the prices of China’s leading stocks

**DOI:** 10.1371/journal.pone.0294460

**Published:** 2023-11-27

**Authors:** Hongli Niu, Qiaoying Pan, Kunliang Xu

**Affiliations:** 1 School of Economics and Management, University of Science and Technology Beijing, Beijing, China; 2 Price Monitoring Center of National Development and Reform Commission, Beijing, China; Central Queensland University, AUSTRALIA

## Abstract

The prediction of stock prices has long been a captivating subject in academic research. This study aims to forecast the prices of prominent stocks in five key industries of the Chinese A-share market by leveraging the synergistic power of deep learning techniques and investor sentiment analysis. To achieve this, a sentiment multi-classification dataset is for the first time constructed for China’s stock market, based on four types of sentiments in modern psychology. The significant heterogeneity of sentiment changes in the sectors’ leading stock markets is trained and mined using the Bi-LSTM-ATT model. The impact of multi-classification investor sentiment on stock price prediction was analyzed using the CNN-Bi-LSTM-ATT model. It finds that integrating sentiment indicators into the prediction of industry leading stock prices can enhance the accuracy of the model. Drawing upon four fundamental sentiment types derived from modern psychology, our dataset provides a comprehensive framework for analyzing investor sentiment and its impact on forecasting the stock prices of China’s A-share market.

## Introduction

In practice, individuals with stronger information processing abilities tend to be more adept at investing [1]. Predicting stock prices using technical indicators has always been a crucial area of research. However, rational economic assumptions do not always align with people’s transaction behavior, as pointed out by Kahneman et al. [2]. This led to the development of behavioral finance, which has conducted several empirical studies demonstrating that investor sentiment is a crucial factor in explaining stock prices [3, 4].

The available research has developed several technical measures of sentiment in the stock market, represented by the BW indicator, calculated by integrating multiple technical indicators [5]. However, the weak-form Efficient Market Hypothesis states that current prices already reflect all historical price and volume data in a mature market, and technical analysis and resulting indicators cannot provide excess returns. In line with this, recent evidence posits that conventional technical measures of sentiment may play a weak role in improving price forecasts and shifts attention to unstructured textual data, such as news and individual comments, generated in real time [6]. The advent of online social networks has resulted in a growing number of investors sharing their comments and sentiments through online investment forums. This has attracted numerous scholars to utilize this information in conjunction with historical stock price data to predict stock price trends [7, 8]. However, most scholars only classify investor sentiment into simple categories, such as positive and negative [9], or neutral and two other categories [10]. This classification is still rudimentary and fails to fully express people’s nuanced sentiments, which may limit the usefulness of subsequent prediction results. To address this issue, some studies on the multi-classification of sentiments in modern psychology [11–13] provide inspiration for more refined sentiment analysis. Furthermore, most studies only rely on news texts for investor sentiment analysis [14, 15]. However, Friesen and Weller [16] argue that due to biases such as conservatism and overconfidence, different investors may interpret the same news differently. Therefore, individual comments can serve as a more direct expression of investor sentiment compared to news articles that are written using standardized language. Therefore, delving deeper into the text-based information derived from stock forums, which captures the personal emotions of individual investors, is a valuable avenue to explore.

When it comes to forecasting the stock market, traditional econometric models can provide some insights into stock market patterns [17–19]. However, due to the complexity of financial data structures, the results of most prediction models fail to satisfy investors. In comparison, AI-based modelling performs better in fitting blue the non-linear correlations and offers significant advantages in stock market forecasting [20]. Among diverse AI algorithms, decision tree-based machine learning models, such as random forest, gradient boosting decision tree and extreme gradient boosting model, perform well in terms of explainability, but deep learning neural networks with more hidden layers and parameters tend to be more powerful in the task of learning non-linear relationships between variables and making predictions [21, 22]. Given that accuracy is the goal that receives the most attention in stock market forecasts, deep learning models are the preferred choice in this work, albeit at the expense of partial explainability. It is worth noting that the Chinese stock market exhibits a unique phenomenon of ups and downs within the stock sector. Stocks belonging to the same sector tend to rise and fall together, while those in different sectors do not show the same trend. Therefore, the difference in investor sentiment is also significant. Most existing studies predict stock prices from the perspective of individual stocks or indices [18, 23]. However, forecasting stock prices in combination with sectors can provide more specific insights for various market participants.

The purpose of this study is to forecast sector stock prices in China by incorporating multiclassification of investor sentiment using deep neural network techniques. Our contribution to the existing literature is twofold. Firstly, this work innovatively starts from the perspective of the industry sector and takes the leading stocks of the industry sector with large volume and high popularity as the prediction object. That is, five leading stocks in the insurance, real estate sector, new energy, consumer sector and the transportation sectors, which are rarely discussed, are selected. Secondly, we construct a sentiment multiclassification dataset for China’s stock market by manually labeling blue the sentiments contained in investor comments on stock forums, using the four categories of sentiments in modern psychology: happiness, anger, fear, and grief. It is utilized to classify investor sentiment of leading stocks in five sectors, and the heterogeneous results of various sectors in the past decade are effectively excavated. Notably, seven commonly-used models in sentiment classification tasks [8, 24] are detailed compared and sorted out on our datasets, including Bi-LSTM, Bi-LSTM-ATT, CNN-Bi-LSTM, CNN-Bi-LSTM-ATT, MULTI-BNN-bow, MULTI-BNN-WORD2VEC and text-CNN (see full names in Table 1).This detailed comparison is novel and has not been previously discussed in literature. It finds that the most suitable investor sentiment classification model for Chinese stock market is Bi-LSTM-ATT. Lastly, the CNN-Bi-LSTM-ATT model [25] is applied to predict prices of five leading stocks based on the constructed sentiment factors and transaction data. The results demonstrate that incorporating the sentiment multi-classification factors into the prediction model has additional predictive power over individual transaction data.

**Table 1:** Abbreviations and their full names.

Abbreviations	Full Name
NLP	Natural Language Processing
ANN	Artificial Neural Network
SVM	Support Vector Machine
RNN	Recurrent Neural Networks
LSTM	Long Short-Term Memory Neural Networks
Bi-LSTM	Bidirectional Long Short-Term Memory Networks
CNN	Convolutional Neural Networks
BNN	Bayesian Neural Networks
ATT	Attention Mechanism
BoW	Bag-of-words model
Bi-LSTM-ATT	Attention-based Bi-LSTM
CNN-Bi-LSTM-ATT	The integrated CNN and Attention-based Bi-LSTM model
Multi-BNN	Multinomial Bayesian Neural Networks
Multi-BNN-BoW	The integrated Multi-BNN and BoW model
Multi-BNN-Word2vec	The integrated Multi-BNN and Word2vec model

The rest of this paper is organized as follows. Section 2 summarizes the latest researches in the field of financial market forecasting and investor sentiment analysis. Section 3 introduces the experimental methods about the text processing, multi-classification of investor sentiment and the forecasting. Section 4 analyzes the experimental results. Section 5 summarizes the findings.

## Literature review

### Stock market forecasting

As an important part of the financial system, the stock market is commonly characterized by high returns and high risks. There has been a lot of literature in academia trying to achieve effective forecasting of stock prices from different perspectives, in which the methods can be categorized into three types, namely, the classical econometric model [17, 18, 26, 27], the machine learning method [28–33], and the deep neural network [25, 34–36]. Since the stock price is always nonlinear, non-stationary, or even seasonally fluctuating, it is difficult to satisfy the basic assumption that the constant variance in the traditional econometric models. Therefore, these econometric models cannot fully catch the complexity of stock price data, and most of the predictions are not ideal.

With the rapid development of artificial intelligence, it has been found that the application of machine learning methods to forecast stock price data does not have many restrictions as the econometric models, and can bring higher forecasting accuracy. For example, Groth and Muntermann [28] applied Naive Bayes, K-Nearest Neighbors, Neural Networks and Support Vector Machines (SVM) for measuring corporate intraday risk exposure levels. Kara et al. [30] utilized two techniques of artificial neural network (ANN) and SVM to predicted the daily rise and fall of the Istanbul National 100 Index. Besides, some works combine machine learning techniques and traditional econometric models to predict financial stock price series [20, 23, 37]. However, the application of machine learning has many limitations. For instance, when the dataset has too much noise or features, it is easy to fall into overfitting. As the sample size increases, the computational complexity will double and some features are not sensitive [38] and the prediction performance will be significantly reduced.

With the vigorous development of deep neural networks, more recently, the research on using this type of method to predict stock prices is constantly enriching. The main neural networks used for stock price predictions include Convolutional Neural Networks (CNN), Recurrent Neural Networks (RNN), Long Short-Term Memory Neural Networks (LSTM), Bidirectional Long Short-Term Memory Networks (Bi-LSTM), Attention Mechanism, etc [34]. For example, Sirignano and Cont [35] applied a 3-layer LSTM to predict the prices of 1000 US stocks in the next day according to the historical orders. Tashiro et al. [39] improved the CNN models (A-CNN, A-CNN+) suitable for capturing order features to predict the short-term trend of stock prices. Besides, scholars formulated hybrid models by combining different neural network models with Attention Mechanisms to predict stock prices. For instance, Chen and Ge [40] integrated the Attention Mechanisms into the LSTM network to improve the movement prediction of 72 stock price in Hong Kong market. Long et al. [25] proposed a deep neural network combining CNN and Attention-based Bi-LSTM model (CNN-Bi-LSTM-ATT) to predict stock price trends using transaction records and public market information. Rezaei et al. [36] combined CNN and LSTM to predict the daily closing prices of the S&P 500, Dow Jones, DAX, and Nikkei 225. The results show that it can improve the prediction accuracy and outperform other baseline models.

However, most of the above studies use the structured data such as stock transaction data, or technical indicators as the input of prediction models, but rarely use unstructured data such as text. Meanwhile, most of the studies ignore the impact of public sentiment on stock price trends. As for prediction objects, most previous studies take the individual stocks and indices as the research objects, but rarely focus on the industry sector. However, in China, there is a rotation between different industries. That is, the ups and downs of the same sector tend to change together with the change of the industry, social phenomena, national policies and the main force’s prediction for the market. For a given period, incremental funds are only concentrated in several hot sectors, and there are huge differences in the performance of stock prices between different sectors, causing significant differences in investor sentiment. Therefore, this paper focuses on combining the investor sentiment and stock transaction data to predict the prices of the leading stocks in five different sectors that have totally different performances, which can provide relevant evidence for researching the development of China’s stock market.

### Investor sentiment analysis on social media

On the basis of the general behavior theory and social network theory [41], information on social media platforms can rapidly disseminate among investors, exerting influence on their expectations, strategies, and perceptions of risk. Subsequently, these factors can reciprocally impact their investment decisions. Substantial empirical evidence suggests that investor sentiment plays a key role in explaining stock price movements [39, 42, 44]. For example, Tetlock [43] reported that the high pessimistic expectations of the media would bring downward pressure on the market prices, and abnormally high or low pessimism heralded a decline in market trading volumes. Hui et al. [44] declared that the investor sentiment extracted from the constructed text information before market opening is helpful to predict the opening price.

Recently, more and more studies have begun to quantify investor sentiment from the textual information on social media. For example, Li et al. [8] classified the sentiments of shareholders of East Money Stock Bar into three categories: positive, negative, and neutral using naive Bayesian classification algorithm. Maqsood et al. [45] classified the words into positive, neutral, and negative categories and calculated the occurrence probability of different types of words in each sentence to judge the sentiment of a tweet. Furthermore, some researchers adopt investor sentiment as an important input variable to predict stock prices. Li et al. [14] projected the main financial news from the Hong Kong Stock Exchange into the constructed sentimental space and combine them with historical prices to forecast the stock prices. Huang et al. [15] classified the sentiment of financial news headlines into two categories, to predict the trend of the Taiwan stock price index. Li et al. [46] classified the sentiment of crude oil news headlines to predict oil futures yield and volatility.

However, most of the existing research using investor sentiment for prediction adopted a simple sentiment classification method. Since the sentiment classification method is too rough, it is difficult to achieve a substantial improvement in forecasting accuracy when introducing the sentiment classification results in stock price prediction. Therefore, considering the complex information contained in the texts, it is necessary to improve the classifying accuracy for investor sentiments when it is used to predict the stock prices.

### Sentiment classification

In modern psychology, various sentiment classification theories were proposed with different opinions [11–13], which are of great significance for our sentimental analysis. For example, Ekman [11] classified sentiments into six basic categories: anger, disgust, fear, happiness, sadness, and surprise. Parrott [12] proposed a tree structure-based model for classifying sentiments including love, happiness, surprise, surprise, anger, sadness, and fear. Plutchik [13] proposed eight basic sentiment categories: grief, fear, surprise, acceptance, ecstasy, rage, vigilance, hatred, and so on. Although scholars have different opinions on the classification of sentiments, modern psychology believes that human beings have only four basic sentiments, namely happiness, anger, fear and grief [47].

Under the above background, several studies on the detailed sentiment classification are provided. For example, by utilizing a manually labeled dataset, Kabir [48] classified the sentiment in Tweets during the Covid-19 into five categories. However, the detailed sentiment classification for textual information in the existing literature mainly focuses on epidemics [48], blogs [49], etc., few works have established a detailed sentiment classification dataset for stock review information on the Chinese stock market. Therefore, in this study, we construct a detailed sentiment classification dataset of the Chinese stock market, and classify the investor sentiment into four categories including happiness, anger, fear and grief [50], so as to extract more sentiment information from texts to improve the stock price prediction.

## Experiment methodology and process

### Experimental design

**Fig 1:**
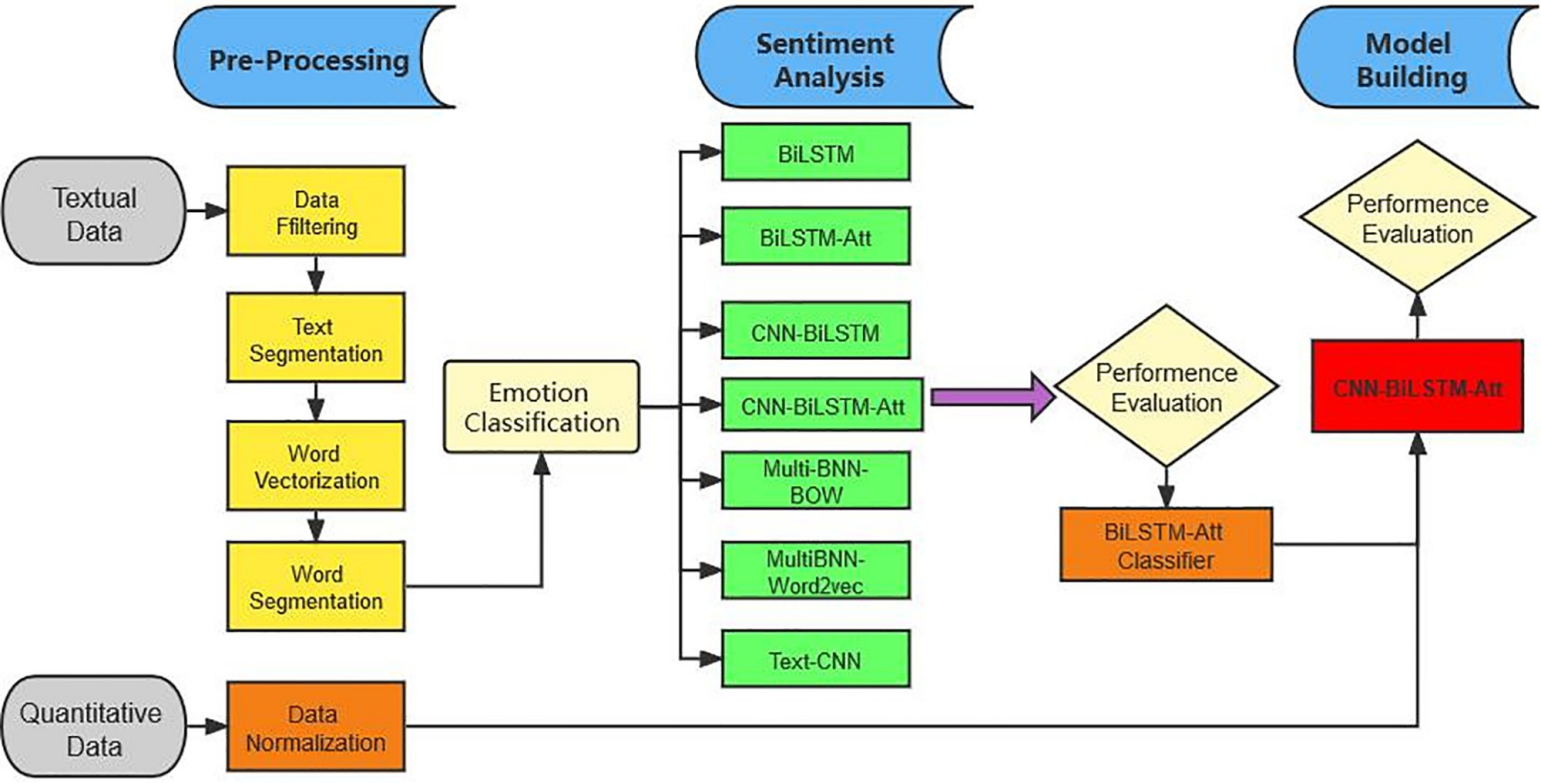
General framework of the proposed model. Note: Three steps are performed in the experiment process. Firstly, pre-processing involves processing textual and quantitative data. The second step is sentiment analysis, various sentiment analysis models such as Bi-LSTM, CNN-Bi-LSTM, CNN-Bi-LSTM-ATT, Multi-BNN-BoW, Multi-BNN-Word2vec, and Text-CNN are compared to identify an effective investor sentiment classification model. In the third model building step, predictions are made on the top five stock prices using the CNN-Bi-LSTM-ATT model. Stock price prediction models with and without investor sentiment are assessed.

In our work, we combine the deep learning methods with the investor sentiment analysis to predict the stock prices of five leading industries in Chinese market. As shown in Fig 1, the experimental process is comprised of three main parts, i.e., data collection and preprocessing, investor sentiment analysis, and stock price prediction modeling.

The first step of the preprocessing involves the processing of both textual and quantitative data. For quantitative data, a data normalization technique is primarily employed. Regarding the textual data, several processes are performed to convert it into machine-understandable form, including data filtering, text segmentation, word vectorization and word segmentation.

In the second step, the aim is to identify an effective investor sentiment classification model. The performances of various models widely acknowledged in previous studies are compared in terms of sentiment analysis. The models considered include Bi-LSTM, Bi-LSTM-ATT, CNN-Bi-LSTM, CNN-Bi-LSTM-ATT, Multi-BNN-BoW, Multi-BNN-Word2vec, and Text-CNN. Multiple indicators are used to assess their performances. The findings indicate that Bi-LSTM-ATT emerges as the most effective model for extracting the investor sentiment.

In the third step, we utilize the CNN-Bi-LSTM-ATT model to make predictions on the stock prices of the five major stocks. To examine whether the inclusion of sentiment indicators can genuinely enhance stock price prediction, two stock price prediction models with and without investor sentiment are compared. Meanwhile, performances of the deep learning method are compared with the benchmark of the Random Walk Method (RW).

### Text preprocessing methods

#### Word segmentation

In the field of natural language processing (NLP), word segmentation technology is essential for the text preprocessing which plays a crucial role in filtering noise from text data and converting it into a machine-understandable format. Jieba, a widely used Python library for word segmentation, is commonly employed in the Chinese NLP applications [50]. Jieba offers four word-segmentation modes: precise mode, full mode, search engine mode, and paddle mode. In this work, the precise mode is chosen as it provides the most accurate sentence segmentation, making it suitable for textual analysis.

Unlike English, where words are typically separated by spaces, Chinese word segmentation requires specific techniques. Therefore, the first step in preprocessing involves splitting sentences into individual words and removing stop words that do not convey essential information, such as “is,” “a,” “on,” “all,” and “the,” among others. In addition to Jieba’s default stop word list, we include stop word lists from Sichuan University Machine Intelligence Laboratory, Harbin Institute of Technology, as well as the Chinese stop word list and Baidu stop word list to enhance the noise reduction process.

Furthermore, to cater to the unique research needs in the financial market, we incorporate the Sogou Financial Thesaurus that is designed for financial industry analysis, to enhance the accuracy of word segmentation. This additional resource supplements Jieba’s built-in thesaurus and ensures better alignment with financial terminology.

Table 2 illustrates the process of Chinese word segmentation by taking a stock review as an example. The first line is the original sentence “Thanks for the generosity of the main force! I’m already full, I’ll see it again at the end of the year, Hahaha!” in English. The second line is the split words, and the last line is the result after deleting the stop words.

**Table 2:** Examples of Chinese text preprocessing.

Type	Data
**Sentence**	感谢主力的慷慨! 我已满仓, 年底再来看, 哈哈哈!
	(Thanks for the generosity of the main force! I’m already full, I’ll see it again at the end of the year, Hahaha!)
**Words**	感谢|主力|的|慷慨|! |我|已|满仓|, |年底|再|来|看|, |哈哈哈|!
	(Thanks for| the generosity |of |the main force|! |I’m| already |full|,| I’ll |see| it|again |at the end of the year|, | Hahaha |!)
**Useful Words**	感谢|主力|慷慨|我|满仓|年底|看|哈哈哈
	(Thanks for| the generosity |the main force |I’m| full| I’ll see| again |at the end of the year | Hahaha |)

#### Word segmentation

Before inputting words into deep learning algorithms, they need to be vectorized. There are generally two methods of word vectorization [51]. The first method is One-hot encoding, which assigns a unique binary representation to each word. The target word is denoted as 1 while other words are represented as 0 in the vector dimensions. Although this method discretizes the data, the resulting vector space tends to be large and features are scattered due to weak semantic connections between words, often leading to dimensional explosion. The second approach is distributed representation. It utilizes low-dimensional and dense word vectors. Each word is mapped to a shorter vector by training a word vector model, enabling the calculation of similarity between word vectors using statistical methods. In this paper, we utilize the Word2vec method [52] released by Google for word vectorization. Word2vec calculates vectors based on the contextual information of words, resulting in a vector space with contextual semantics measured by the angle and distance between words. This model allows semantically similar words to cluster together in the word vector space.

For training the Word2vec model, we employ a Chinese corpus obtained from Wikipedia. Given a sentence with *n* words, the Word2vec method generates an *n* × *k* two-dimensional matrix, where *k* represents the dimension of the word vector. Typically, *k* ranges between 100 and 800. Table 3 provides an example of word vectors for a sentence, where each word is mapped to a vector based on the corpus. The cosine similarity between the vectors reflects the degree of similarity between the words.

**Table 3:** Examples of Chinese text preprocessing.

Words	d1	d2	…	dk
感谢(Thanks for)	0.38	0.29	…	0.92
主力(the main force)	0.71	0.35	…	0.84
慷慨(the generosity)	0.54	-0.72	…	0.75
我(I’m)	-0.12	0.51	…	0.65
满仓(full)	0.47	0.76	…	0.24
年底 at the end of the year	-0.39	1.22	…	-0.33
看 see	0.28	-0.77	…	-0.99
哈哈哈 (Hahaha)	0.67	0.82	…	0.56

### Sentiment multi-classification method

According to the four basic categories of sentiments in modern psychology, namely happiness, anger, grief, and fear, this paper constructs a text dataset suitable for the sentiment multi-classification of the Chinese stock market. In the multi-classification task, sentiment labels are usually annotated manually. In this work, we collect 8,195 stock reviews from Eastern Fortune (https://guba.eastmoney.com) and manually label them into four categories of sentiments. To avoid being too subjective, we invite three professors specializing in finance to manually label each comment with the sentiment. When the three agree on the sentiment label contained in the same sentence, the label is determined as the sentiment of the sentence. If there is a difference, judge the sentiment according to the principle of the minority obeying the majority. If the three people had completely different labels, they would redo the labeling action and repeat the above principles until the results came out. According to the statistics of the dataset, there are 2083 happy stock reviews, 2004 angry stock reviews, 2049 grief stock reviews, and 2059 angry stock reviews. To mention, the comments used in this study are posted publicly and anonymously by commentators. Meanwhile, the information on the platform is publicly accessible. Therefore, this study does not address any potential ethical considerations such as data privacy and user consent. Table 4 lists several manually sentiment classification results of stock comment.

**Table 4:** Examples of Chinese text preprocessing.

Comments	Type	Number
坐好要飞了[呲牙] 额,下午应该会涨停吧哈哈哈 已经赚了一倍哈哈哈!	happiness	2083
我要气死了 真想割肉真垃圾 大垃圾退市吧	anger	2004
已经亏抑郁了 伤心太平洋 每天都幻想大涨,每天都失望	grief	2049
莫追小心有雷,这票太诡异 快跑,要跌了 好吓人	fear	2059

### Sentiment analysis model Bi-LSTM-ATT

**Fig 2:**
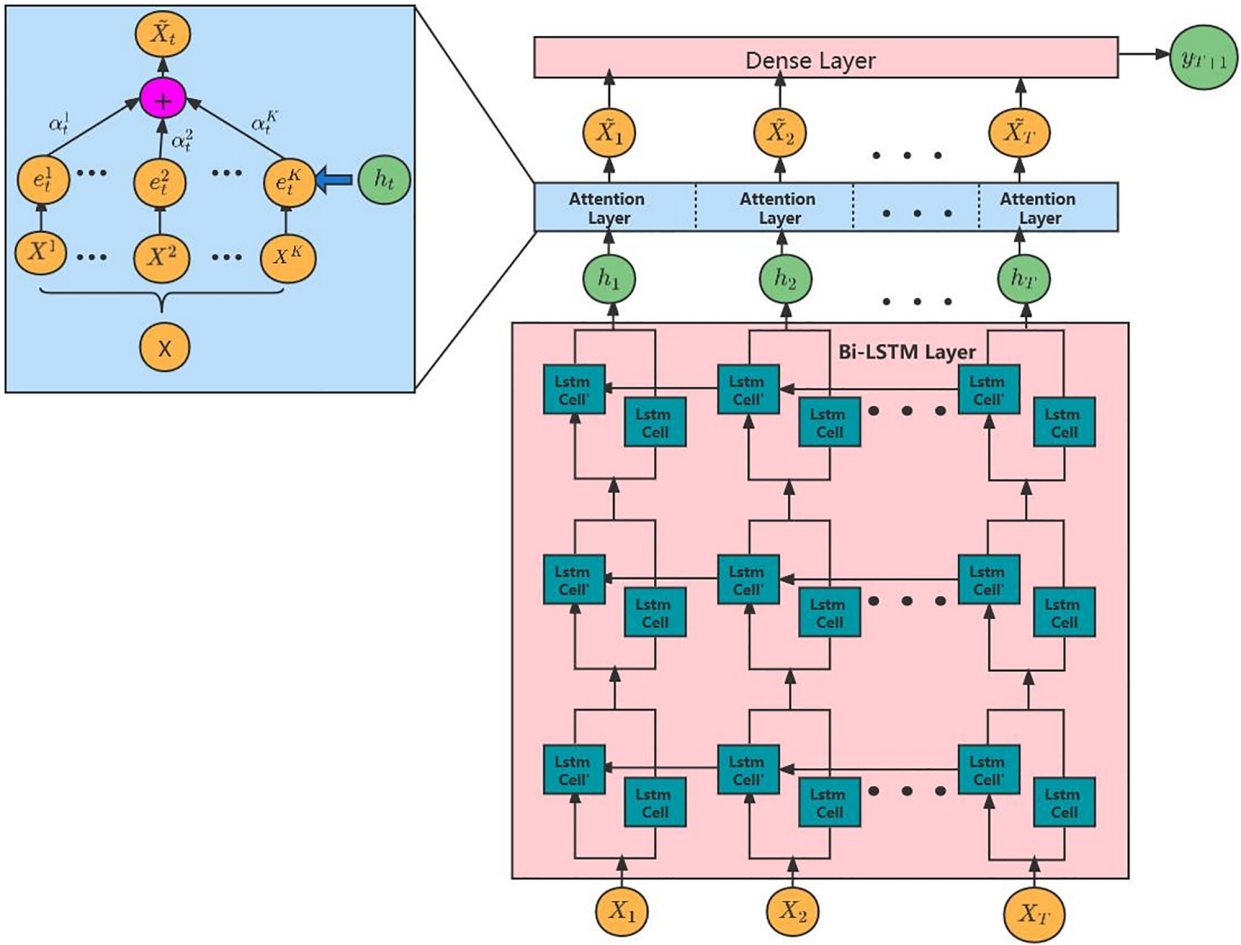
The framework of the proposed model Bi-LSTM-ATT. Note: The Bi-LSTM-ATT classifier consists of an input layer, three Bi-LSTM layers, an attention layer and a fully connected layer. *X*_1_, *X*_2_, ⋯, *X*_*T*_ represents a sentence has *T* words, LSTM cells represents basic LSTM cells, which capture past and future information. In the attention layer, the difference between different features can be calculated, by which we can tell the degree of importance and assign corresponding weights to the information.

In recent years, the excellent performance of hybrid models in classifying investor sentiment has been widely proved [10]. Therefore, the Bi-LSTM-ATT model, which is widely used in the fake news detection [53] and text-classification [54], is employed in this work for sentiment analysis. The model is formulated by integrating bidirectional LSTM and Attention Mechanism, enabling it to effectively capture sequential information and discern disparities among feature data. By emphasizing the transmission of more significant information, the model exhibits improved predictive performance, especially for longer time series. The Bi-LSTM-ATT classifier consists of an input layer, three Bi-LSTM layers, an attention layer and a fully connected layer, as is shown in Fig 2.

Assuming that a sentence has *T* words after word segmentation and stop word removal, a *K* × *T* matrix is obtained after processing with word vectors. The number of rows *K* is the dimension of the word vector, and the number of columns *T* is the number of words in a sentence. Transpose the vector on input. Then the input vector is expressed as: *X* = [*X*_1_ *X*_2_ ⋯ *X*_*T*_]_1*T_, $X_{t}=\left(x_{t}^{1},x_{t}^{2},\cdots ,x_{t}^{K}\right)\in R^{K}$ represents a vector containing *K* features at time *t*. Set the vector y as the target vector. Consequently, the target of our work is to learn a nonlinear mapping:

$$\begin{array}{c} y=f(X_{1},\cdots ,X_{T}), \end{array}$$ (1)

where *f*(⋅)is the nonlinear mapping function.

Bi-directional LSTM was introduced by Hochreiter and Schmidhuber [55], which is refined and popularized in many works such as context-dependent natural language processing [56]. It is composed of two basic LSTM cells, one is a forward LSTM conveying past information and another one is a reverse LSTM conveying future information. Therefore, at time t, the information of time *t* − 1 and time *t* + 1 can be used simultaneously. In general, Bi-LSTM is better than LSTM in time series prediction because each sequence is presented forward and backward as two independent hidden states that capture past and future information, respectively. For the input word vector sequence **X** = (*X*_1_, *X*_2_, ⋯ *X*_*T*_), where $X_{t}\in R^{K}$, *K* represents the number of features in the sequence data, the Bi-LSTM network learns the mapping from *X*_*t*_ to *h*_*t*_:

$$\begin{array}{c} h_{t}=f_{1}\left(h_{t-1},X_{t}\right) \end{array}$$ (2)

*h*_*t*_ is the hidden layer vector at time t, and *f*_1_(⋅) is a recurrent neural network unit. The first layer of Bi-LSTM abstracts the implicit information contained in each series from the input data. The next two layers are utilized to extract more in-depth information on the basis of the previous layer, and later the implicit information will be used in the computation of the Attention Mechanism.

Next, the Attention Mechanism is added [57]. Essentially, Attention Mechanism is a method for output Y to assign different attention to different sections of input X, which is defined as the contribution or weight of each section of *X*. By adding Attention Mechanism to the model, we can capture the difference between different features, calculate their degree of importance and assign corresponding weights to them. As a result, the time vector *X*_*t*_ is able to choose and deliver more important information adaptively. For the input data sequence *X* = (*X*^1^, *X*^2^, ⋯, *X*^*K*^)^*T*^, a feature-based Attention model is constructed in the Bi-LSTM-ATT model as follows:

$$\begin{array}{c} e_{t}^{i}=V^{T}\;\text{tanh}\left(Wh_{i}^{\prime }+UX^{i}+b\right) \end{array}$$ (3)

$$\begin{array}{c} \alpha_{i}^{l}=\frac{\text{exp}(e_{t}^{i})}{\sum_{j=1}^{K}\text{exp}\left(e_{t}^{j}\right)} \end{array}$$ (4)

where $V^{T}\in R^{T},W\in R^{T*hidden_{size,}},U\in R^{T*T}$ is the weight matrix to be learned, $b\in R^{T}$ is the bias vector to be learned, $\alpha_{t}^{i}$ is the attention weight of the *i*-th feature at time *t*, which represents the importance of $x_{t}^{i}$ in $X_{t}=\left(x_{t}^{1},x_{t}^{2},\ldots ,x_{t}^{K}\right)\in R^{K}$. The softmax function in the equation makes sure that the sum of all attention weights equals 1. The result is utilized to assign weights to the input vector *X*_*t*_ at time *t*.

$$\begin{array}{c} {\tilde{X}}_{t}=\left(\alpha_{t}^{1}x_{t}^{1},\alpha_{t}^{2}x_{t}^{2},\cdots ,\alpha_{t}^{N}x_{t}^{N}\right) \end{array}$$ (5)

After the attention layer, ${\tilde{X}}_{t}$ selectively focuses on different features instead of distribute equal weight to each feature compared to *X*_*t*_. Then ${\tilde{X}}_{t}$ will be combined with $h_{t}^{{}^{\prime }}$ to get the output value *a*_*t*_ of the attention layer:

$$\begin{array}{c} a_{t}=f_{2}\left({\tilde{X}}_{t},h_{t}^{{}^{\prime }}\right)=\text{tanh}\left(W_{a}\left[{\tilde{X}}_{t};h_{t}^{{}^{\prime }}\right]\right) \end{array}$$ (6)

where $h_{t}^{{}^{\prime }}$ is the output of the previously hidden layer. Therefore, the model not only pays attention to the information of the entire sequence but also adaptively focuses on the information of different features according to the importance at time *t*. Finally, *a*_*t*_ becomes the input data of the dense layer of the Bi-LSTM-ATT.

The last layer of Bi-LSTM-ATT is the dense layer, which takes the output vector *A*_*t*_ = (*a*_1_, *a*_2_, …, *a*_*T*_) of the attention layer as input and learns the transfer function from *A*_*t*_ to *y*. That is, through the last dense layer, *y* is the final result of sentiment classification.

$$\begin{array}{c} y=f_{3}\left(W_{y}*A_{t}+b_{y}\right) \end{array}$$ (7)

where *f*_3_(⋅) is the SoftMax function in the last dense layer of the Bi-LSTM-ATT, *W*_*y*_ is the weight matrix, and *b*_*y*_ is the bias vector.

### Stock prediction model CNN-Bi-LSTM-ATT

**Fig 3:**
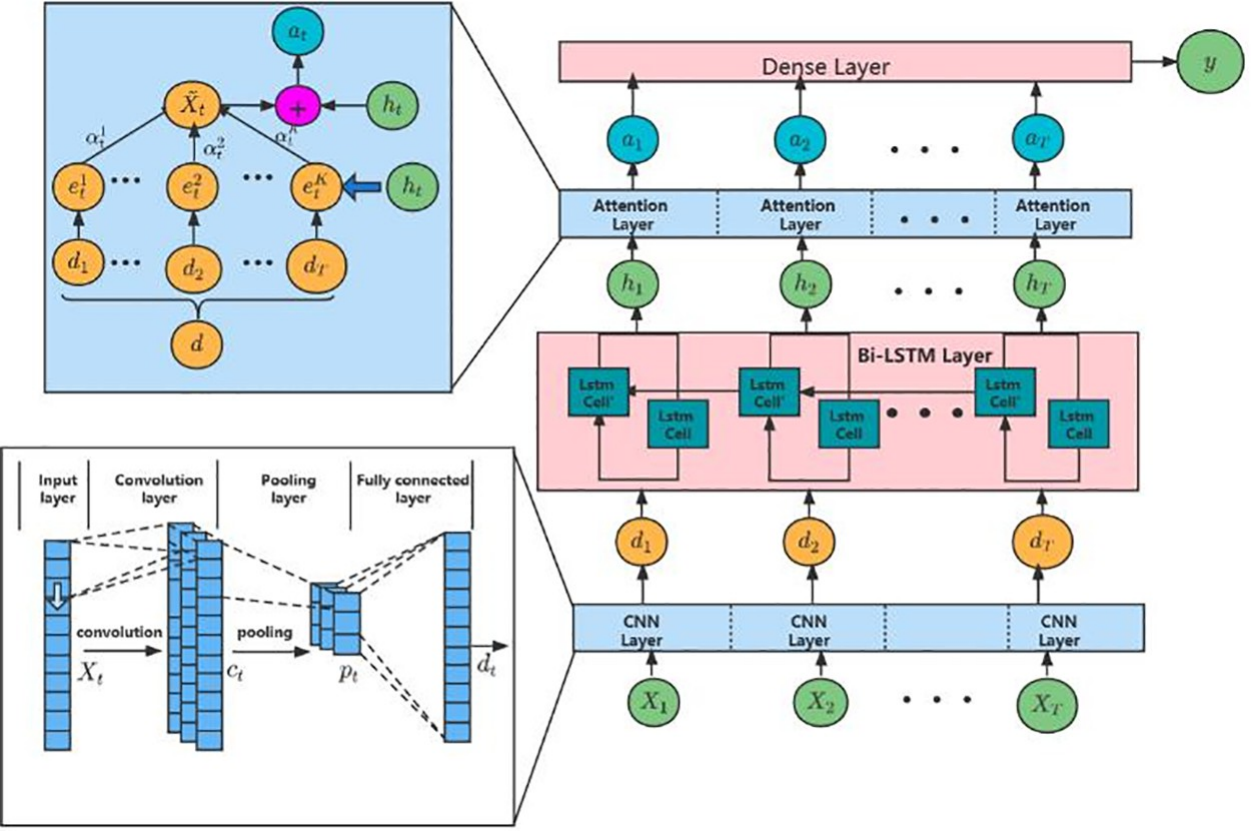
The framework of the proposed model CNN-Bi-LSTM-ATT. Note: CNN-Bi-LSTM-ATT classifier comprises multiple layers. It starts with an input layer followed by a CNN layer. *X*_1_, *X*_2_, ⋯, *X*_*T*_ represents groups of time series.Three Bi-LSTM layers are then employed to capture past and future information. The attention layer plays a crucial role in calculating the differences between various features. This calculation enables the determination of feature importance, allowing for the assignment of corresponding weights to the information. Finally, a fully connected layer is utilized in the classifier.

In our work, the CNN-Bi-LSTM-ATT model [28] is applied to forecast the closing prices of five leading stocks in different industry sectors. The model has a significant advantage over traditional models in robustness and practicality for stock price prediction. It is composed of connecting the Bi-LSTM neural network after the CNN neural network and adding a layer of Attention Mechanism, which can not only capture the implicit data features from the original dataset via CNN but also learn the contextual information from Bi-LSTM. Its structure is displayed in Fig 3.

For the input stock time series **X** = (*X*_1_, *X*_2_, ⋯ *X*_*T*_), where $X_{t}\in R^{N}$, *N* means the groups of time series to describe stock information. First, the raw data is fed into the CNN model, then calculated by the convolution layer, activation function (ReLU) and max-pooling layer and drop-out layer, and then the final output vector is calculated as follows:

$$\begin{array}{c} c_{t}=f_{4}\left(w_{t}*X_{t}+b_{t}\right) \end{array}$$ (8)

$$\begin{array}{c} \gamma \left(c_{t},c_{t-1}\right)=\text{max}\left(c_{t},c_{t-1}\right) \end{array}$$ (9)

$$\begin{array}{c} p_{t}=\gamma \left(c_{t},c_{t-1}\right)+\beta_{t} \end{array}$$ (10)

$$\begin{array}{c} d_{t}=f_{2}\left(t_{t}p_{t}+\delta_{t}\right) \end{array}$$ (11)

where *d*_*t*_ represents the final output vector of CNN, *δ*_*t*_ is the bias vector to be learned, and *t*_*t*_ is the weight matrix to be learned. Next, the result of feature extraction through the convolutional layer is input into the Bi-LSTM layer.

## Evaluation indicators

### Indicators for sentiment classification task

To evaluate the effects of sentiment multi-classification task by the deep neural network, the commonly-used evaluation metrics, Precision, Recall, F1-Score and Accuracy are adopted. F1-Score, also known as Balanced Score, is defined as the harmonic mean of precision and recall. “Accuracy” represents the proportion of the sample correctly classified among all samples. They are defined as follows:

$$\begin{array}{c} Precision=\frac{TP}{TP+FP} \end{array}$$ (12)

$$\begin{array}{c} Recall=\frac{TP}{TP+FN} \end{array}$$ (13)

$$\begin{array}{c} F_{1}-score=2\cdot \frac{precision\cdot recall}{precision+recall} \end{array}$$ (14)

$$\begin{array}{c} Accuracy=\frac{TP+TN}{TP+TN+FP+FN} \end{array}$$ (15)

where TP and TN represent “True Positive” and “True Negative”, representing the number of positive samples and negative samples that are correctly predicted respectively. FP and FN stand for “False Positive” and “False Negative”, which denote the total number of samples that are incorrectly predicted as positive and negative, respectively. The better the model can classify the sentiment, the higher value of Precision, Recall, F1-score and Accuracy are.

To comprehensively test the classification effect of the model on various sentiments, two more concepts, Macro avg and Weighted avg, are introduced, which are calculated as follows:

$$\begin{array}{c} \text{Macro}\;\text{avg}=\frac{\sum_{i=1}^{k}\nu_{i}}{k} \end{array}$$ (16)

$$\begin{array}{c} \text{Weighted}\;\text{avg}=\frac{\sum_{i=1}^{k}\nu_{i}n_{i}}{\sum_{i=1}^{k}n_{i}} \end{array}$$ (17)

where the *ν*_*i*_ represents the value of the *i*-th emotion for one indicator, *k* represents the type of the emotions, and *n*_*i*_ represents the number of samples of the *i*-th emotion.

### Indicators for stock forecasting task

To examine the ability of CNN-Bi-LSTM-ATT for stock price predictions, several metrics in Table 5 are introduced to evaluate the results, including Mean Squared Error (MSE), Root Mean Squared Error (RMSE), Mean Absolute Error (MAE), Mean Absolute Percentage Error (MAPE), Mean Squared Logarithmic Error (MSLE) and *R*2_*score* in measuring the level of prediction error. The better predictive performance of the model, the smaller MSE, RMSE, MAE, MAPE, MSLE, and the larger *R*2_*score*.

**Table 5:** Commonly-used performance evaluation metrics.

$MSE=\frac{1}{n}\sum_{i=1}^{n}{\left({\hat{y}}_{i}-y_{i}\right)}^{2}$	$MSLE=\frac{1}{n}\sum_{i=1}^{n}{\left(\text{log}\left({\hat{y}}_{i}+1\right)-\text{log}\left(y_{i}+1\right)\right)}^{2}$
$RMSE=\frac{1}{n}\sqrt{\sum_{i=1}^{n}{\left({\hat{y}}_{i}-y_{i}\right)}^{2}}\sum_{i=1}^{n}{\left({\hat{y}}_{i}-y_{i}\right)}^{2}$	$R2\_score=1-\frac{\sum_{i}{\left({\hat{y}}_{i}-y\right)}^{2}}{\sum_{i}{\left(\bar{y}-y_{i}\right)}^{2}}$
$MAE=\frac{1}{n}\sum_{i=1}^{n}\left|{\hat{y}}_{i}-y_{i}\right|$	$MAPE=\frac{1}{n}\sum_{i=1}^{n}\left|\frac{{\hat{y}}_{i}-y_{i}}{y_{i}}\right|$

Note: *n* is the data length, *y*_*t*_ is the real data, and ${\hat{y}}_{t}$ is the predicted data.

Moreover, the Diebold-Mariano (DM) test [58] is usually used to investigate the null hypothesis of the forecasting precision of model_2 equals its benchmark model_1 in the sense of statistics. The DM statistic can be calculated as:

$$\begin{array}{c} DM=\frac{\bar{d}}{{\left({\hat{V}}_{\bar{d}}/T\right)}^{1/2}} \end{array}$$ (18)

where

$$\begin{array}{c} \bar{d}=1/T\sum_{t=1}^{T}\left[{\left(x_{t}-{\hat{x}}_{1,t}\right)}^{2}-{\left(x_{t}-{\hat{x}}_{2,t}\right)}^{2}\right] \end{array}$$ (19)

$$\begin{array}{c} {\hat{V}}_{\bar{d}}=\gamma_{0}+2\sum_{l=1}^{\infty }\text{cot}\left(d_{t},d_{t-1}\right) \end{array}$$ (20)

${\hat{x}}_{1,t}$ and ${\hat{x}}_{2,t}$ represents the predicted value of model_1 and model_2 respectively. The results of the DM test need to comprehensively consider the DM value and the p-value. When DM > 0, it means that model_2 outperforms model_1 under the significance level of 1 − *p*, and vice versa.

## Experiments and results

### Data collection and preprocessing

The prices of the leading stocks in five different industry sectors of Chinese stock market are predicted in this paper. Based on the comprehensive references of the total stock market value, total share capital, the number of A-shares in circulation, net profit, operating income, and the number of shareholders, the selected leading stocks include Ping An (601318) in the insurance sector, Vanke (000002) in the real estate sector, BYD (002594) in the new energy sector, Kweichow Moutai (600519) in the consumer sector, and Shanghai Airport (600009) in the transportation sector. The various indicators of the five stocks are shown in Table 6. The time period is from November 27, 2011 to November 27, 2021. Some stocks may be temporarily suspended due to some special situations such as major information disclosure, clarification, and announcement of major issues, etc., so the trading date of each stock will be slightly different, as shown in Table 5 where the data lengths excluding non-trading time are given. Besides, many basic indicators are often used to predict market trends and stock prices [23]. In this paper, we select the quantitative indicators of daily opening price, closing price, highest price, lowest price, and trading volume of the stock as parts of the model input. All datasets are from WIND database.

**Table 6:** Examples of Chinese text preprocessing.

Stock code	Total market value	Total share capital	Circulating A-shares	Net profit	Total operating income	Number of shareholders	Data point
601318	9678	182.8024	108.3266	1430.99	12183	130.7854	2428
000002	2576	116.2538	97.1755	415.1554	4191.1168	53.8847	2277
002594	7170	29.1114	11.5506	42.3427	1565.9769	36.0177	2420
600519	24697	12.562	12.562	466.9729	979.9324	17.5267	2428
600009	963	19.2696	10.9348	-12.6665	43.0347	28.9634	2420

**Fig 4:**
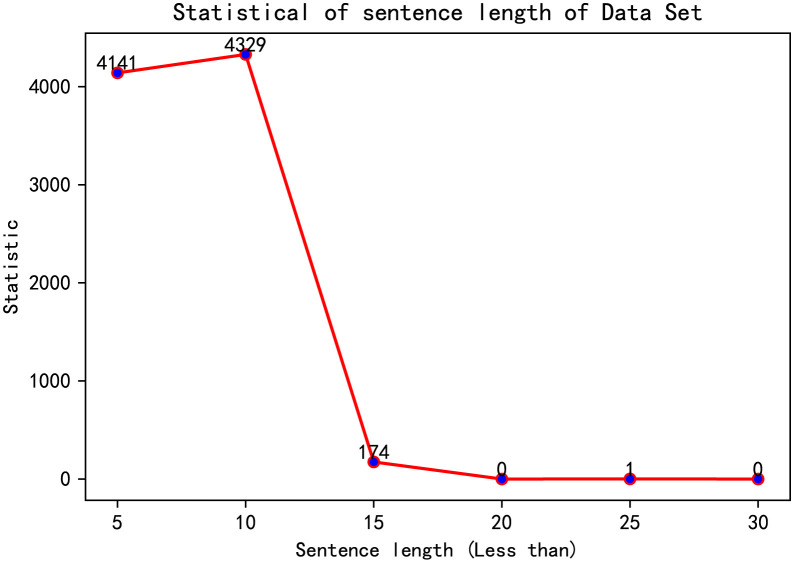
Statistical results of the length of stock comments. Note: the sentence lengths of data set consisting of 8,195 popular reviews are calculated to align the length of the input sequence.

For the sentiment multi-classification task, a Python-based web crawler is designed to extract stock comments from Eastern Fortune Forum. In this work, totally 8,195 popular reviews are obtained, in which most of them are short sentences, and more than 99% of the sentences are less than 20 words in length. Fig 4 displays the statistical results of the length of stock reviews. Therefore, in the word vector processing for stock reviews, we uniformly set the length of the word vector to 20. In order to align the length of the input sequence, if the length of the text is less than 20, a zero vector is used to fill it to 20; if the length of the text is greater than 20, it is clipped to 20. The analysis of investor sentiment is based on the most read stock reviews of the five stocks as the proxy of investors’ sentiment on that day.

To reduce the influence of noise and differences in the size of different datasets on the training results and to optimize the training process, all the input data is normalized to the range of [0, 1] as follows:

$$\begin{array}{c} x{\left(t\right)}^{\prime }=\frac{x(t)-\text{min}\;x(t)}{\text{min}\;x(t)-\text{min}\;x(t)} \end{array}$$ (21)

The normalized data is then fed into the neural network. In order to visually compare the predicted results with the true values, we denormalize the output as follows:

$$\begin{array}{c} x\left(t\right)=x{\left(t\right)}^{\prime }\left(maxx\left(t\right)-\text{min}\;x\left(t\right)\right)+\text{min}\;x\left(t\right) \end{array}$$ (22)

### Results of sentiment multi-classification

To identify the investor sentiment more accurately, we constructed a textual classification dataset of investor sentiment on the basis of the sentiment classification in modern psychology, namely happiness, anger, grief, and fear. It is utilized to train several different sentiment analysis models that are widely recognized in the previous studies [8, 27], including Bidirectional LSTM (Bi-LSTM), Bi-LSTM-ATT, CNN-Bi-LSTM, CNN-Bi-LSTM-ATT, Multi-BNN-BoW, Multi-BNN-Word2vec, and Text-CNN, and the performances of these models in sentiment analysis are compared. In order to control variables and fairly compare the performance of sentiment analysis between different models, we apply the same initial parameters to all the above models. Through repeated experiments, the parameters of several models including the final best Bi-LSTM-ATT are determined, as shown in Table 7.

**Table 7:** The initial parameters of several models including the Bi-LSTM-ATT.

Hyperparameter	Description	Range
Vocab Dim	Dimension of word vector	100
Learning Rate	How much to change the model in response to the estimated error	0.001
Learning Rate Decay	How much does the learning rate decrease in each round of training	0.00001
Epochs Size	The number of times that the neural network passes through the entire training	25
Batch Size	The number of samples that will be propagated through the neural network	15

**Fig 5:**
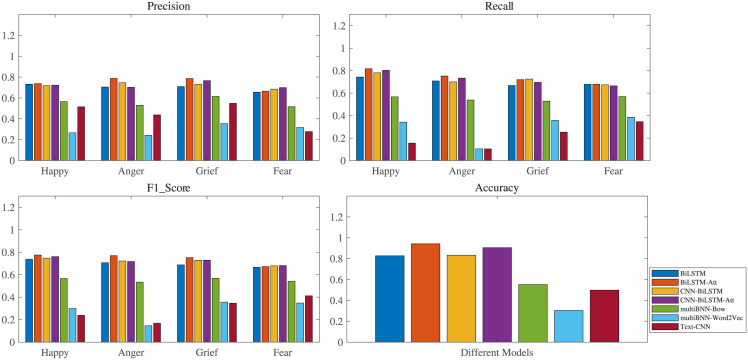
The effects of different models on the four sentiment classifications. Note: the Bi-LSTM-ATT classifier outperforms other models in sentiment analyses.

Fig 5 shows the prediction effects of different models for each sentiment type separately. It is observed that whether from the indicators of Precision, Recall, *F*_1_-score or Accuracy, the effects of Multi-BNN-BoW, Multi-BNN-Word2vec and Text-CNN models are significantly worse compared with the other models combined with Bi-LSTM. Among them, the sentiment classification accuracy obtained by the two Multi-BNN models are even lower than 50%, which shows that the Bi-LSTM is a key factor to improve the accuracy in sentiment classification. In addition, after adding the Attention Mechanism, both the Bi-LSTM-ATT model and CNN-Bi-LSTM-ATT models obtain a higher classification effect, and the Attention Mechanism improves the effect of the Bi-LSTM model in the sentiment classification task more significantly. Moreover, in all models, the Bi-LSTM-ATT classifier has the highest Recall value in the classification of sentiment for happy, anger and fear, the highest Precision value for happy, anger and grief, the highest *F*_1_-score value for happy, anger and grief, and the highest Accuracy value, which demonstrates that the Bi-LSTM-ATT classifier outperforms other models in sentiment analyses.

The results of Macro avg and Weighted avg are shown in the Tables 8 and 9. For the Bi-LSTM-ATT sentiment classification model, the macro average values of Precision, Recall and F1-score reach 74.412%, 74.234%, and 74.224% respectively, which all outperform those of other models. Besides, the weighted average values of Precision (74.306%), Recall (74.104%) and F1-score (74.105%) of Bi-LSTM-ATT model are also the largest. These further indicate that the Bi-LSTM-ATT model has significant advantages over other models in sentiment classification tasks, and is a feasible tool to analyze the sentiments hidden in text data. Therefore, this work applies the trains Bi-LSTM-ATT to classify the sentiment of unlabeled text of leading stocks in five different industries of Chinese stock market, which are used for the subsequent prediction task.

**Table 8:** Macro avg results.

Model	Precision	Recall	*F*_1_-score
Bi-LSTM	0.70069	0.69945	0.69986
Bi-LSTM-ATT	0.74412	0.74234	0.74224
CNN-Bi-LSTM	0.71959	0.71998	0.71924
CNN-Bi-LSTM-ATT	0.72216	0.72445	0.72215
Multi-BNN-BoW	0.55646	0.55104	0.55260
Multi-BNN-Word2vec	0.29462	0.29727	0.28699
Text-CNN	0.44464	0.32806	0.29117

**Table 9:** Weighted avg results.

Model	Precision	Recall	*F*_1_-score
Bi-LSTM	0.69828	0.69780	0.69782
Bi-LSTM-ATT	0.74306	0.74104	0.74105
CNN-Bi-LSTM	0.71775	0.71747	0.71711
CNN-Bi-LSTM-ATT	0.72152	0.72065	0.71996
Multi-BNN-BoW	0.55509	0.55176	0.55224
Multi-BNN-Word2vec	0.29681	0.30336	0.29160
Text-CNN	0.44384	0.33642	0.29518

**Fig 6:**
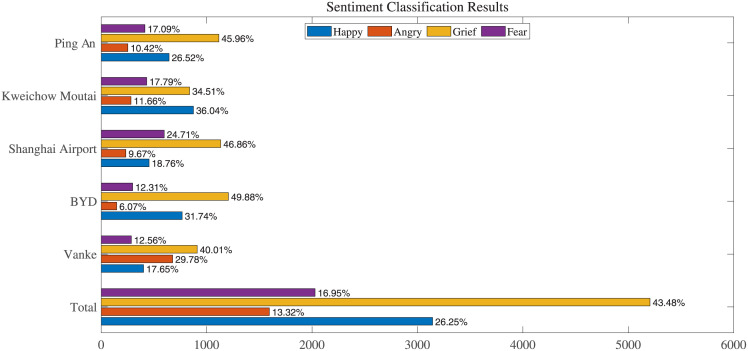
The effects of different models on the four sentiment classifications. Note: the duration of sentiments has exhibited significant variations across industries throughout the past decade (2011–2021). However, a consistent pattern emerges where the grief sentiment has consistently prevailed for the longest duration over the ten-year period. Conversely, the market sentiment related to fear has exhibited relatively shorter durations across all five industries, indicating that despite experiencing various sectors’ stock prices going through both long and short corrections over the past decade, the Chinese stock market has seldom fallen into a state of panic.

The statistical results of the sentiment classification of stocks in five industries are exhibited in Fig 6, showing that the duration of all sentiments varies significantly across industries over the past decade (2011–2021). For the stocks in the consumer sector represented by Kweichow Moutaiv (600519), the happy sentiment lasted for the longest period of time among the four sentiments, reaching 36.04% of the number of trading days, which can be explained by the continuous rise in the share price of the consumer sector in the past ten years to a certain extent. Simultaneously, the high expectations of the market have also played a certain role in promoting the rise of the share price, the two complement each other and form a positive cycle. Besides, the new energy sector represented by BYD (002594) and the financial sector represented by Ping An (601318) are also seen as more optimistic by the market for a long period of 768 and 644 trading days respectively, which is highly related to the country’s policy background of vigorously developing new energy industry and financial industry. Compared with other sectors, although the stocks in the real estate sector represented by Vanke (00002) have the shortest time to make investors feel happy, it is worth noting that they also make investors feel fearful for a relative shorter period of time. This shows that in the past decade, although the investors have always maintained a dissatisfied attitude towards the trend of real estate stocks, but in reality, people have always had a certain degree of confidence in its future, a short-term share price cut will not plunge the people into complete despair. By contrast, the duration in which investors feel fearful for the trend of stock price is the longest in the transportation industry represented by Shanghai Airport (600009) among all other sectors, accounting for 24.71% of all trading dates, which is close to a quarter. This has a huge correlation with the disruption of flights under the influence of the epidemic, which reflects that due to the long-term impact of the COVID-19 epidemic, investors are full of worries and doubts about the development of the transportation industry.

A common feature of the investors’ sentiment in different industry sectors is that the grief sentiment has accounted for the longest period of time in the past ten years, reaching an average of 43.48% of the trading days in the five leading stocks. On the one hand, this matches the characteristics of Chinese stock market where the bull market cycles are short but the bear market cycles are long, on the other hand, it is seen that when the stock market is not optimistic, investors may be more willing to share their emotions on platforms such as stock forums for comfort and resonance. In addition, the market sentiment about fear has a relatively shorter duration in all the five different industries, only reaching an average of 16.95% of the trading days, which may indicate that although the stock prices of various sectors in the Chinese stock market have undergone long or short corrections over the past decade, investors’ expectations for the long-term development of the stock market have remained positive, so the market rarely falls into panic.

### Results of stock price forecasting

In this section, we make predictions on the five leading stock prices by using the CNN-Bi-LSTM-ATT model. To illustrate whether the introduction of the sentiment indicators can indeed improve the stock price prediction, two experiments are conducted. In the first experiment, marked as model_1, only the trading data of each stock is taken into the CNN-Bi-LSTM-ATT model to perform the forecasting. In the second experiment, labeled as model_2, both the trading data and the sentiment indicators are simultaneously considered as the input. Additionally, to strengthen the persuasiveness of the analysis and mitigate potential misleading results solely based on error evaluation indicators, this section incorporates a non-statistical learning method known as the Random Walk Method (RW). The RW method utilizes the current period’s price as a benchmark to predict the next period’s price. It is used for comparison with the deep learning method, allowing for the calculation and comparison of prediction errors between the two approaches. The parameters of the CNN-Bi-LSTM-ATT model are determined by trial and error, which are given in Table 10. During the experiment, the data samples are divided into three sets: 70% of the samples were allocated as the training set, 10% as the cross-validation set, and the remaining 20% as the test set.

**Table 10:** The initial parameters of the CNN-Bi-LSTM-ATT model.

Hyperparameter	Description	Range
Test Size	The proportion of the data used in the training set	0.2
Validation Split	The proportion of the data used in the cross-validation set	0.1
Learning Rate	How much to change the model in response to the estimated error	0.01
Time Steps	The length of data used to predict the stock price for the next day	20
Epochs Size	The number of times that the neural network passes through the entire training	100
Batch Size	The number of samples that will be propagated through the neural network	64

Table 11 presents the error evaluation metrics for the baseline model, model_1, and model_2. It is evident that both model_1 and model_2 outperform the baseline model across most of the error evaluation indicators. Notably, metrics such as MSE, RMSE, MAE, MAPE, and MSLE exhibit significantly higher values in the baseline model compared to the CNN-Bi-LSTM-ATT models. This observation highlights the effectiveness of the CNN-Bi-LSTM-ATT model in predicting stock prices. Furthermore, when comparing model_2 with model_1, most of the error evaluation indicators show a significant decrease. This indicates that the inclusion of sentiment indicators positively contributes to the prediction performance of CNN-Bi-LSTM-ATT across various industries. Specifically, the MAPE values in model_2 are substantially lower than those in model_1 for all stocks analyzed. Taking BYD stock (002594) as an example, the MSE, RMSE, and MAE values in model_1 without sentiment indicators are 273.601, 16.541, and 9.200, respectively. However, after incorporating sentiment factors, the MSE decreases to 90.134, the RMSE decreases to 9.494, and the MAE decreases to 6.010 in model_2.

**Table 11:** The evaluation metrics of model_1 (without sentiment), model_2 (with sentiment) and benchmark model.

Stocks	Models	MSE	RMSE	MAE	MAPE	R2_Score	MSLE
**000002**	**model_1**	2.219	1.490	1.003	5.559	0.970	0.005
	**model_2**	1.767	1.329	0.909	4.761	0.976	0.004
	**benchmark**	11.718	13.693	2.053	6.225	0.750	6.017
**002594**	**model_1**	273.601	16.541	9.200	16.384	0.934	0.047
	**model_2**	90.134	9.494	6.010	11.971	0.978	0.026
	**benchmark**	8.965	11.977	17.438	13.721	0.510	2.506
**600009**	**model_1**	8.952	2.992	2.203	7.036	0.982	0.007
	**model_2**	5.854	2.420	1.659	4.855	0.988	0.004
	**benchmark**	10.357	12.873	1.903	13.697	0.630	3.984
**600519**	**model_1**	4758.8	68.984	45.086	9.463	0.987	0.016
	**model_2**	2009.1	44.823	30.551	7.465	0.995	0.011
	**benchmark**	116.612	43.195	50.778	51.990	10.210	2.809
**601318**	**model_1**	10.217	3.196	2.302	4.363	0.973	0.004
	**model_2**	6.450	2.540	1.772	3.217	0.983	0.002
	**benchmark**	9.108	12.072	17.668	17.907	0.520	2.380

**Fig 7:**
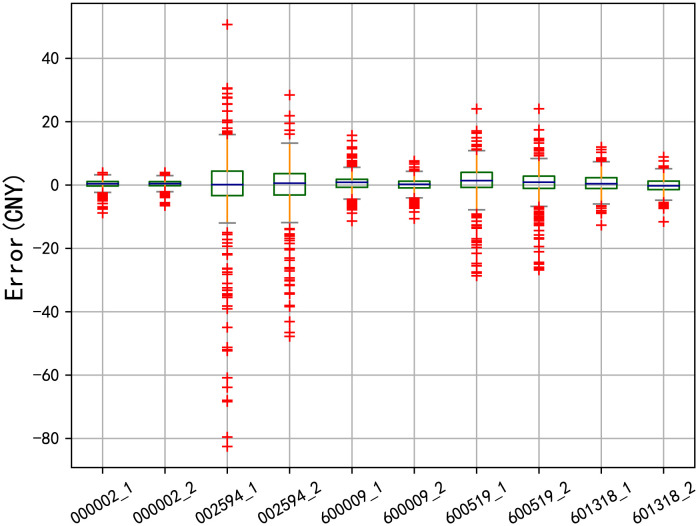
The boxplots of errors between the model with and without sentiment indicators. Note: The box height of model_2 is noticeably smaller compared to that of model_1, suggesting that the inclusion of sentiment indicators enhances the prediction accuracy of the model.

Fig 7 displays the boxplots of bias between the stock predicted series and the true value. It shows the solid lines in the boxes of all the five stocks are near the abscissa, indicating that the median error of the model either with or without the sentiment indicators is near zero, that is, the prediction error is generally small and the prediction effect is good. Besides, the height of the box of model_2 is significantly shorter than that of model_1, which means the difference between the upper and lower quartiles of error sequence in model_2 is reduced and the distribution of its error values is more concentrated around zero. It indicates that the prediction precision of the model is enhanced when the sentiment indicators are added.

The DM test is further presented in Table 12 to verify the superiority of model_2 against model_1 from a statistical point. The *p*-values of all five stocks are much less than the significant level of 1%, which means that the CNN-Bi-LSTM-ATT model with the sentiment indicators has a statistically better forecasting effectiveness under the confidence level of 99%. In other word, this again confirms the necessity of considering market sentiment factors in stock forecasting.

**Table 12:** DM test results.

	000002	002594	600009	600519	601318
**DM**	3.2087	6.1931	9.7675	8.0041	7.3813
***p*-value**	1.43E-03	1.33E-09	1.46E-20	1.04E-14	7.66E-13

## Conclusion

A large number of empirical results indicate that investor sentiment is a key factor in explaining stock price trends, and the rise of online social networks has accelerated the spread of investor sentiment. To better study the impact of changes in investor sentiment on the stock price and improve the accuracy of stock price prediction results, this paper combines blue the deep learning methods with the sentiment analysis to predict the prices of leading stocks in popular industries in China’s A-share market.

Firstly, a sentiment multi-classification text dataset with four kind of emotions for the Chinese stock market is constructed, according to the sentiment classification in modern psychology with the investor sentiment manually classified. Several common sentiment analysis models are trained and compared to find the best deep learning model for investor sentiment classification. It is verified that the Bi-LSTM-ATT model is superior with a comparison with other 6 models including Bi-LSTM, CNN-Bi-LSTM, CNN-Bi-LSTM-ATT, Multi-BNN-BoW and Multi-BNN-Word2vec and text-CNN. Through mining the market sentiment, it is found that over the past decade, there have been significant differences in the investor sentiment among different industries, and the reasons for these differences are influenced by the comprehensive development of the industry itself and blue the national policy guidance. The investors have been optimistic about the stocks from the consumer sector represented by Kweichow Moutai (600519) for the longest period of time. This is partly related to the continuous rise of share prices in the consumer industry in the past decade. In turn, the high expectations of the market also played a certain role in promoting the rise of share prices. The new energy sector represented by BYD (002594) and the financial sector represented by Ping An (601318) are also more optimistic by the market for a longer period of time. The happy mood lasted 768 trading days and 644 trading days respectively, which is highly related to the policy background of the country to vigorously develop the new energy industry and the financial industry. In contrast, investors have always maintained a dissatisfied attitude towards the trend real estate sector represented by Vank (000002) and are full of worries and doubts about the long-term development of the transportation industry represented by Shanghai Airport (600009). A common feature of the investors’ sentiment in different industry sectors is that the duration of sad sentiment has accounted for the longest period of time in the past ten years, which is consistent with the short bull market cycle and long bear market cycle of the Chinese stock market.

Then, the sentiment indicators and stock daily transaction data are taken to predict the leading stock prices of five sectors by the CNN-Bi-LSTM-ATT neural network. The comparison experiment with and without the sentiment indicators are performed respectively. The findings indicate that the CNN-Bi-LSTM-ATT model with market sentiment factors does reduce the error of stock price prediction and improve the prediction accuracy.

Further, we must mention that though the comment of investors in the Eastern Fortune Forum is very representative in the judgment of investor sentiment, they do not provide a comprehensive portrait of the market. The dataset’s construction may be constrained by time and geographic location. Moreover, this study only considers emotions and historical stock data as the variables for predicting stock prices, disregarding the influence of other potential factors on stock prices, simplifying the multifaceted nature of investment decisions. In the future work, there are several avenues for enhancing the robustness of this research. For example, analyzing investor sentiment using a broader range of text sources like online financial news, newspaper articles, chat messages, research reports and financial statements. Besides, investigating additional factors contributing to varying stock price fluctuations among industries, such as industry cycles, macroeconomic influences, policy shifts, and geopolitical factors, can offer valuable avenues for future research.

## Supporting information

S1 Data (ZIP)
