# Supplementary figures and images for: Hybrid deep learning models with multi-classification investor sentiment to forecast the prices of China’s leading stocks
Source: PLoS One. 2023 Nov 27;18(11):e0294460. doi: 10.1371/journal.pone.0294460 (PMC10681238; doi:10.1371/journal.pone.0294460)

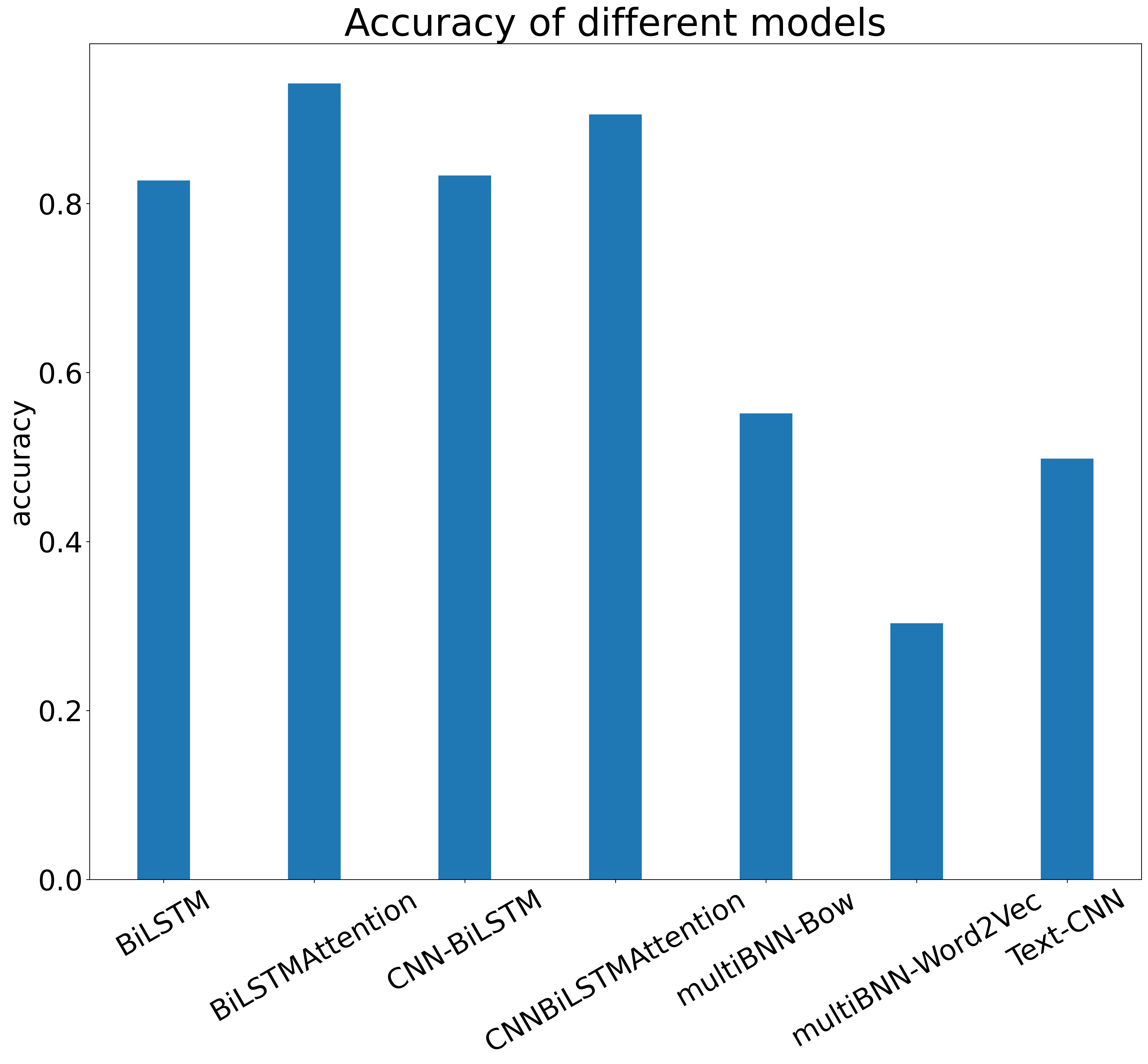

Supplement: S1 Data — (ZIP) [file pone.0294460.s001.zip › code and data/data processing -python/5:word2vec+sentiment classification/pictures/Comparison in different prediction models/accuracy.png]

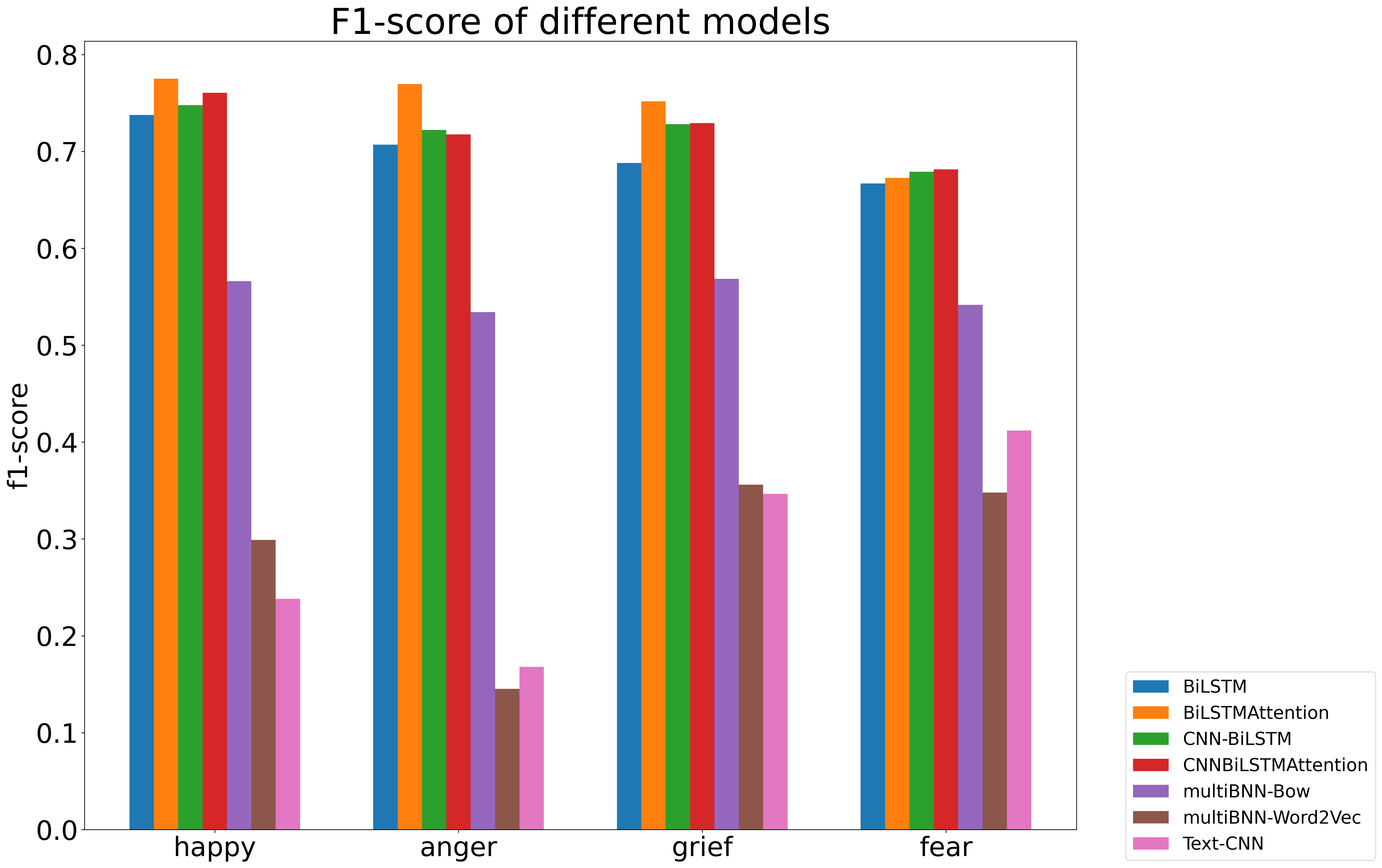

Supplement: S1 Data — (ZIP) [file pone.0294460.s001.zip › code and data/data processing -python/5:word2vec+sentiment classification/pictures/Comparison in different prediction models/f1-score.png]

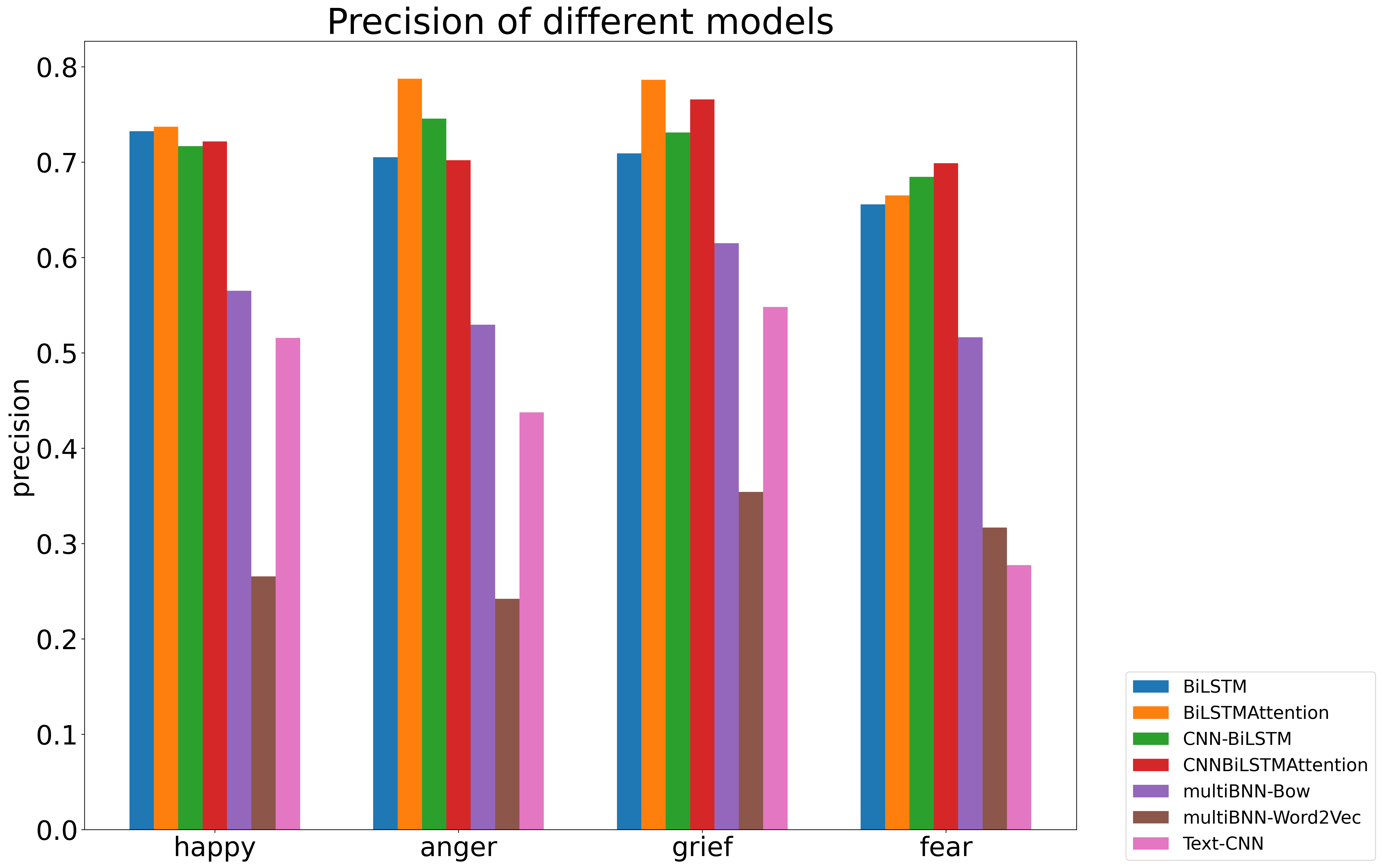

Supplement: S1 Data — (ZIP) [file pone.0294460.s001.zip › code and data/data processing -python/5:word2vec+sentiment classification/pictures/Comparison in different prediction models/precision.png]

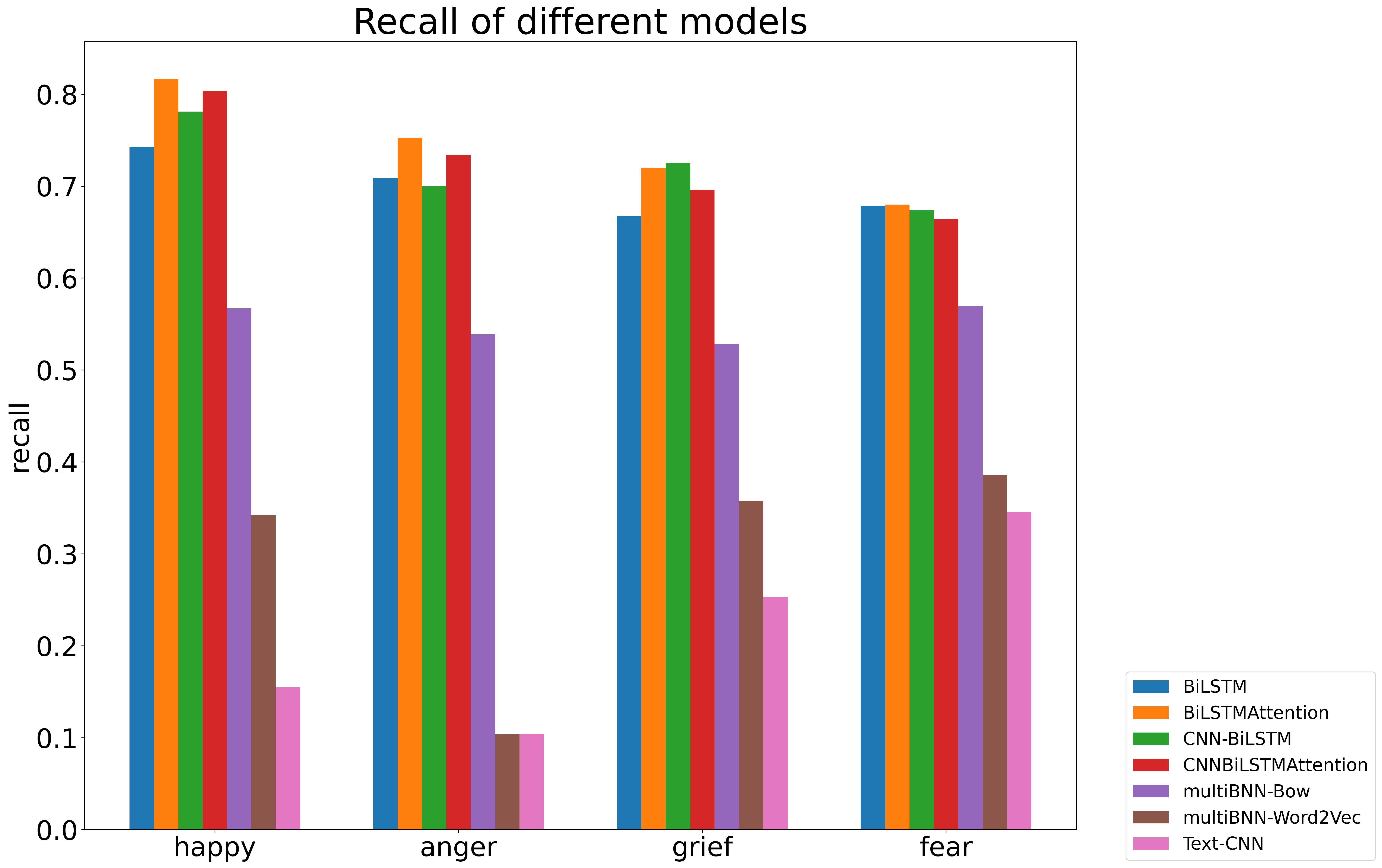

Supplement: S1 Data — (ZIP) [file pone.0294460.s001.zip › code and data/data processing -python/5:word2vec+sentiment classification/pictures/Comparison in different prediction models/recall.png]

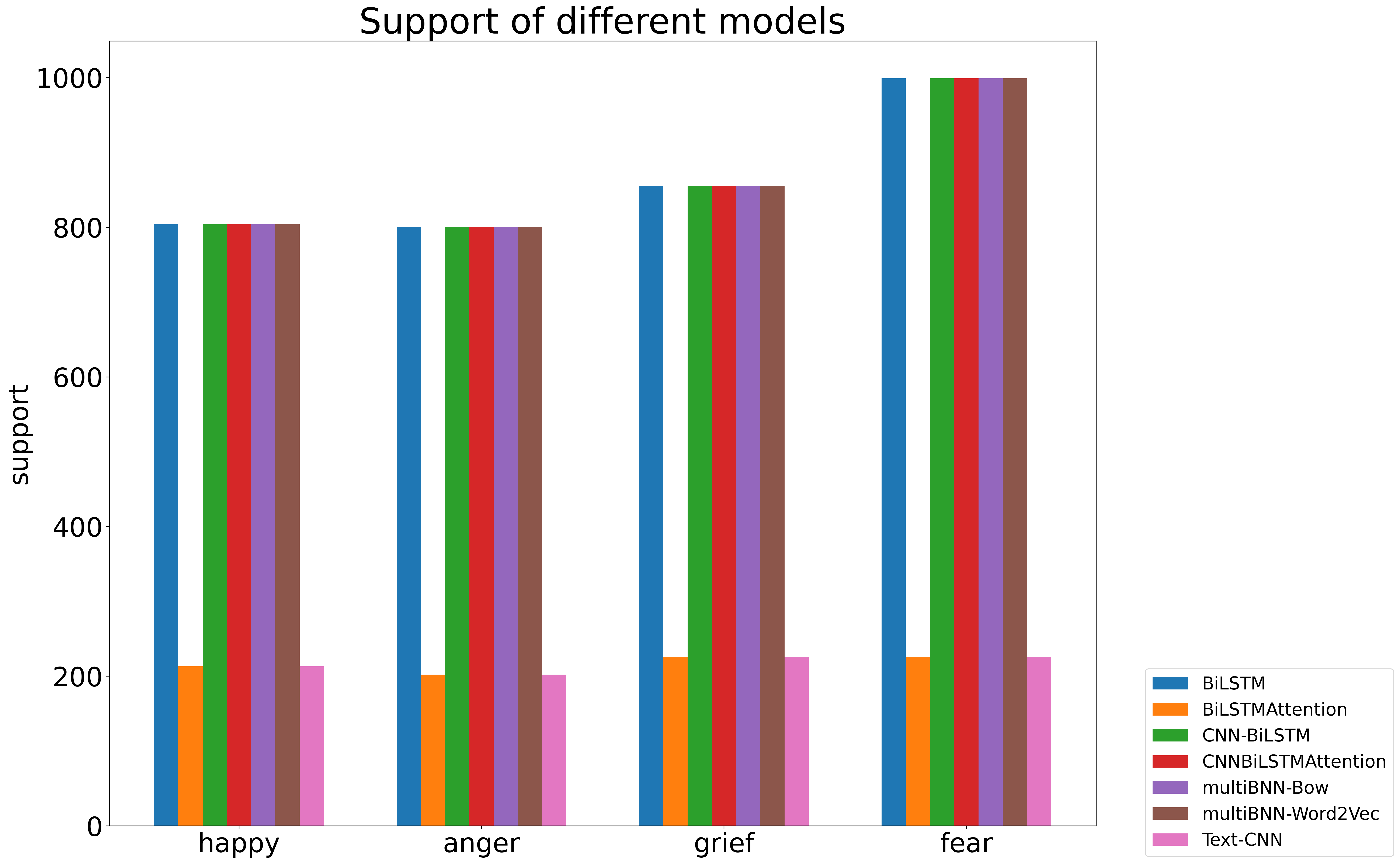

Supplement: S1 Data — (ZIP) [file pone.0294460.s001.zip › code and data/data processing -python/5:word2vec+sentiment classification/pictures/Comparison in different prediction models/support.png]

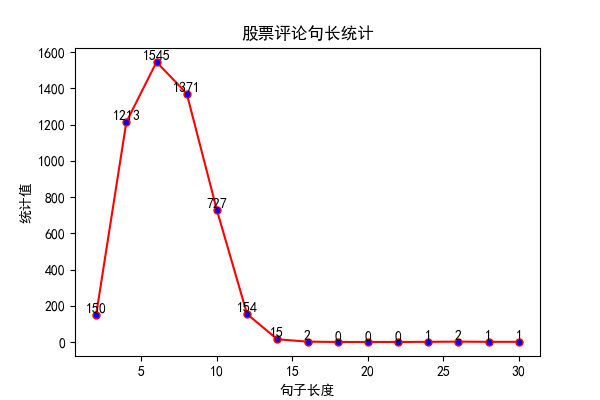

Supplement: S1 Data — (ZIP) [file pone.0294460.s001.zip › code and data/data processing -python/5:word2vec+sentiment classification/pictures/Statistical of sentence length of Data Set.png]

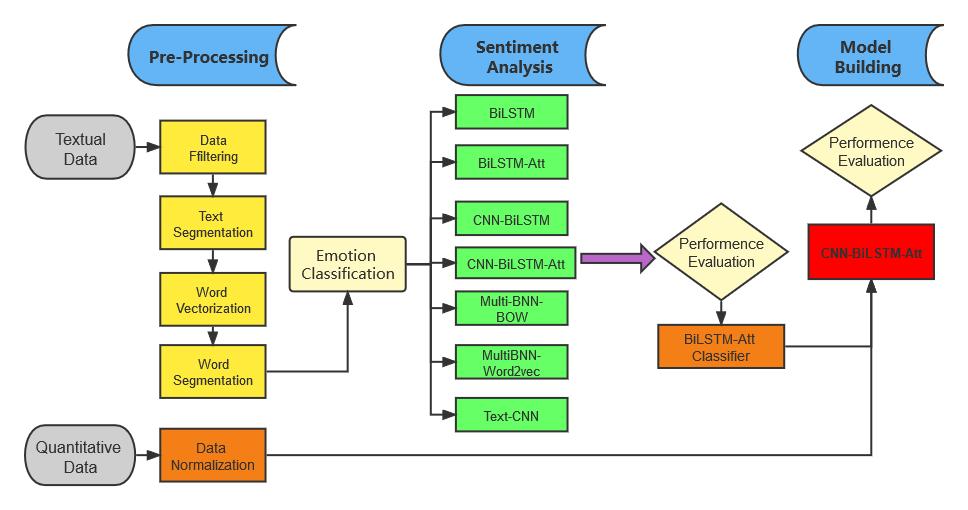

Supplement: S1 Data — (ZIP) [file pone.0294460.s001.zip › code and data/Figure/Figure 1.jpg]

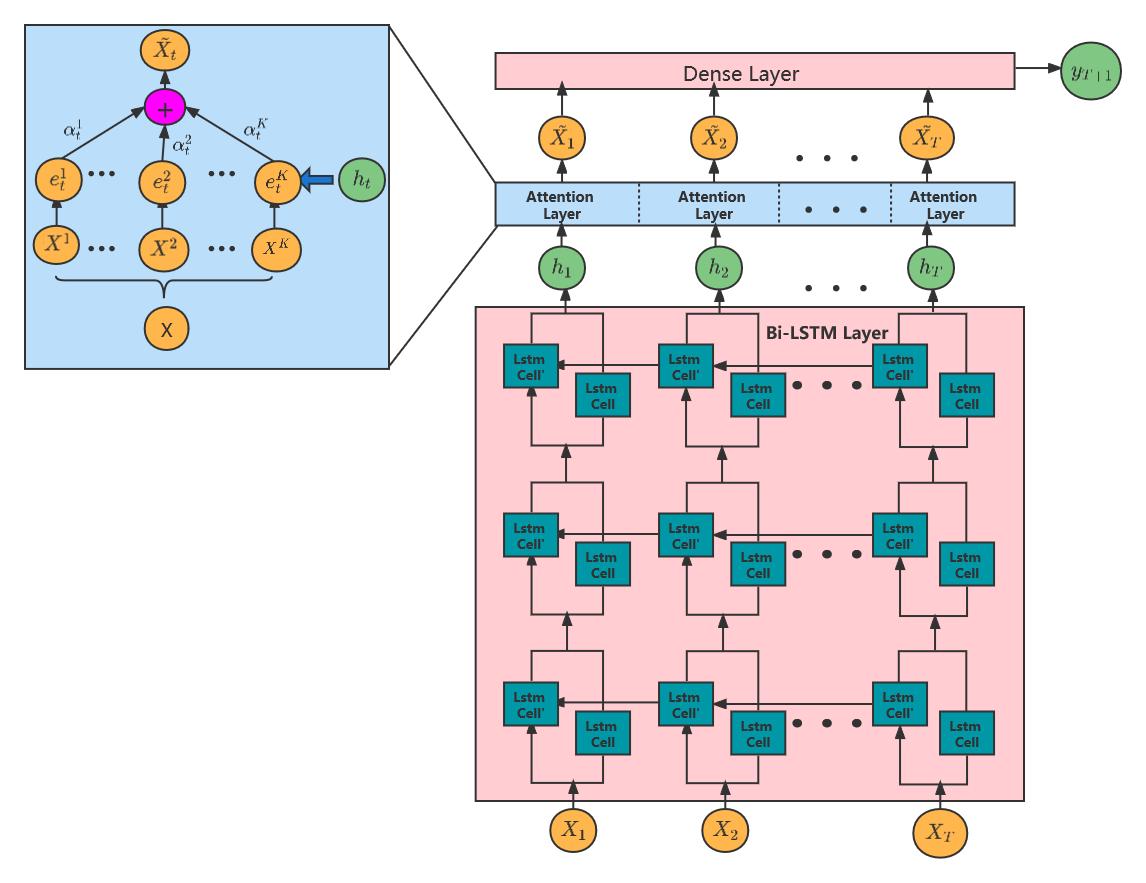

Supplement: S1 Data — (ZIP) [file pone.0294460.s001.zip › code and data/Figure/Figure 2.jpg]

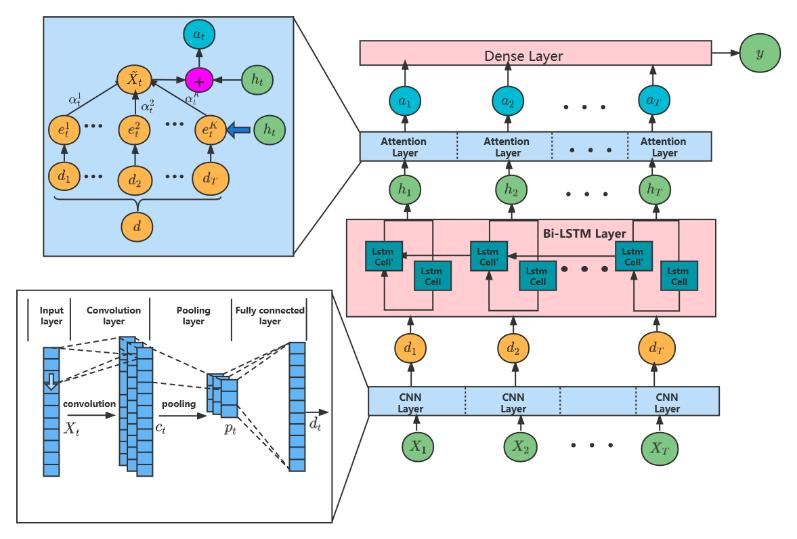

Supplement: S1 Data — (ZIP) [file pone.0294460.s001.zip › code and data/Figure/Figure 3.jpg]

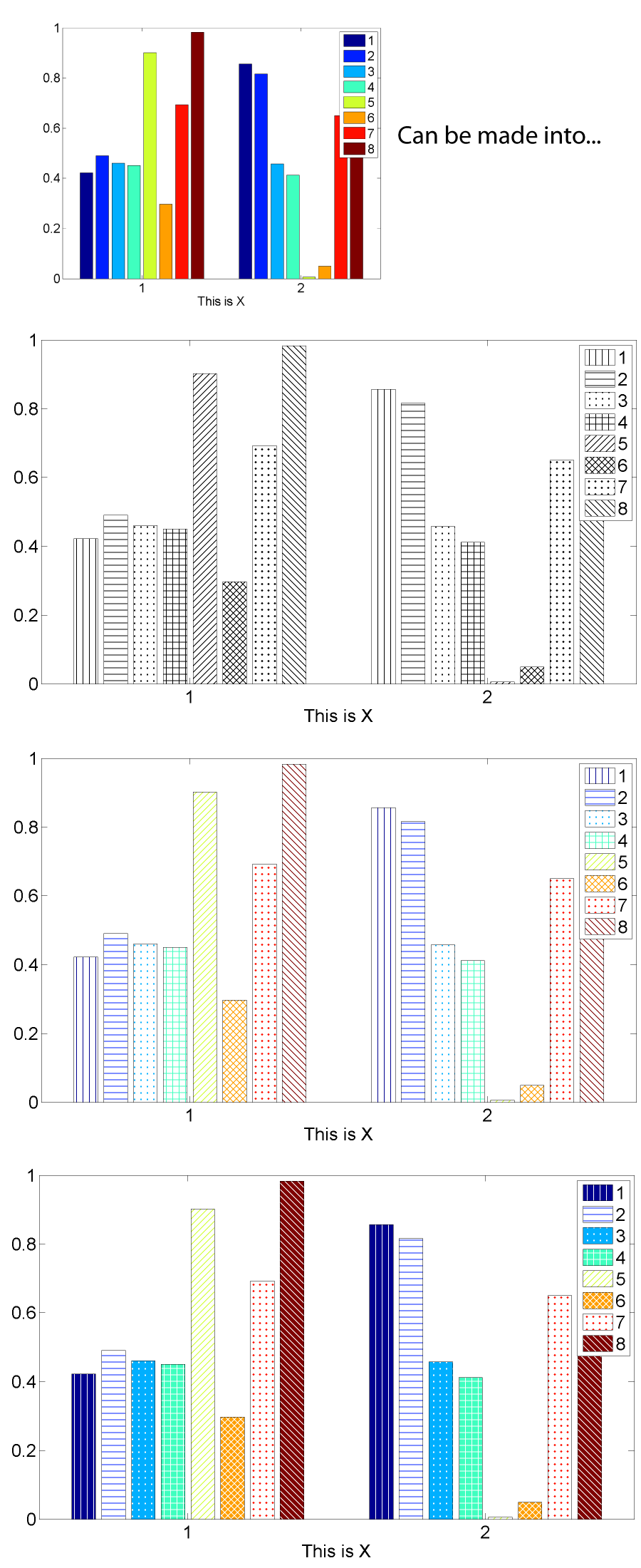

Supplement: S1 Data — (ZIP) [file pone.0294460.s001.zip › code and data/figure coding-matlab/figure_demo/Figure_applyhatch/applyhatch_pluscolor_image.png]
